# Supplementary material for: Development and evaluation of a lightweight large language model chatbot for medication enquiry
Source: PLOS Digit Health. 2025 Sep 4;4(9):e0000961. doi: 10.1371/journal.pdig.0000961 (PMC12410746; doi:10.1371/journal.pdig.0000961)
Supplement: S1 Table — (DOCX) [file pdig.0000961.s001.docx]

S1 Table: List of Medications included in Fine-tuning Dataset

| Medication List | |
| --- | --- |
| 1 | Acetaminophen |
| 2 | Acetylcysteine |
| 3 | Alfacalcidol |
| 4 | Alfuzosin |
| 5 | Allopurinol |
| 6 | Amitriptyline |
| 7 | Amlodipine |
| 8 | Atenolol |
| 9 | Azathioprine |
| 10 | Baclofen |
| 11 | Betahistine |
| 12 | Bethanechol |
| 13 | Calcitriol |
| 14 | Calcium acetate |
| 15 | Calcium carbonate |
| 16 | Canagliflozin |
| 17 | Carbamazepine |
| 18 | Carbidopa and levodopa |
| 19 | Carbimazole |
| 20 | Carvedilol |
| 21 | Clonazepam |
| 22 | cotrimoxazole |
| 23 | Cyanocobalamin |
| 24 | Cyclosporine |
| 25 | Dapagliflozin |
| 26 | Donepezil |
| 27 | Dulaglutide |
| 28 | Dutasteride |
| 29 | Empagliflozin |
| 30 | Enalapril |
| 31 | Entecavir |
| 32 | Escitalopram |
| 33 | Esomeprazole |
| 34 | Ezetimibe |
| 35 | Famotidine |
| 36 | Ferric derisomaltose |
| 37 | Ferrous gluconate |
| 38 | Fexofenadine |
| 39 | Fluoxetine |
| 40 | Fluvoxamine |
| 41 | Folic acid |
| 42 | Gabapentin |
| 43 | Gliclazide |
| 44 | Glimepiride |
| 45 | Glyburide |
| 46 | Hydralazine |
| 47 | Hydrocortisone |
| 48 | Hydroxychloroquine |
| 49 | Hydroxyzine |
| 50 | Insulin degludec |
| 51 | Insulin detemir |
| 52 | Insulin glargine and lixisenatide |
| 53 | Insulin glargine |
| 54 | Insulin glulisine |
| 55 | Insulin lispro |
| 56 | Insulin NPH and insulin regular |
| 57 | Ketoprofen |
| 58 | Lamotrigine |
| 59 | Lanthanum |
| 60 | Levetiracetam |
| 61 | Levodopa and benserazide |
| 62 | Levothyroxine |
| 63 | Linagliptin and metformin |
| 64 | Liraglutide |
| 65 | Loperamide |
| 66 | Loratadine |
| 67 | Mesalamine |
| 68 | Methotrexate |
| 69 | Mirtazapine |
| 70 | Montelukast |
| 71 | Morphine |
| 72 | Mycophenolate mofetil |
| 73 | Orphenadrine |
| 74 | Pancrelipase |
| 75 | Phenytoin |
| 76 | Pioglitazone |
| 77 | Prednisolone |
| 78 | Pregabalin |
| 79 | Propranolol |
| 80 | Pyridostigmine |
| 81 | Quetiapine |
| 82 | Repaglinide |
| 83 | Rosiglitazone |
| 84 | Rosuvastatin |
| 85 | Selegiline |
| 86 | Semaglutide |
| 87 | Senna |
| 88 | Sertraline |
| 89 | Sevelamer |
| 90 | Simvastatin |
| 91 | Sitagliptin and metformin |
| 92 | Sodium bicarbonate |
| 93 | Sodium chloride preparations |
| 94 | Sodium polystyrene sulfonate |
| 95 | Solifenacin |
| 96 | Spironolactone |
| 97 | Sulfasalazine |
| 98 | Tacrolimus |
| 99 | Tamsulosin |
| 100 | Telmisartan |
| 101 | Tolbutamide |
| 102 | Tramadol |
| 103 | Trihexyphenidyl |
| 104 | Ursodeoxycholic acid |
| 105 | Valproate |
| 106 | Valsartan |
| 107 | Vildagliptin |
| 108 | Vitamin C |
| 109 | Vitamin D3 |
| 110 | Warfarin |
