# Supplementary material for: Development and evaluation of a lightweight large language model chatbot for medication enquiry
Source: PLOS Digit Health. 2025 Sep 4;4(9):e0000961. doi: 10.1371/journal.pdig.0000961 (PMC12410746; doi:10.1371/journal.pdig.0000961)
Supplement: S2 Table — The fine-tuning dataset comprised of medications from various ATC categories. We display ATC categories represented in our fine-tuning dataset, from ATC level 1 to level 4. (DOCX) [file pdig.0000961.s002.docx]

S2 Table: Anatomical Therapeutic Chemical (ATC) Class. The fine-tuning dataset comprised of medications from various ATC categories. We display ATC categories represented in our fine-tuning dataset, from ATC level 1 to level 4.

| **ATC Level 1** | **ATC Level 2** | **ATC Level 3** | **ATC Level 4** |
| --- | --- | --- | --- |
| Alimentary Tract and Metabolism | Antidiarrheals, intestinal antiinflammatory / Antiinfective Agents | Intestinal antiinflammatory agents | Corticosteroids acting locally |
|  | Antidiarrheals, intestinal antiinflammatory / Antiinfective Agents | antipropulsives | antipropulsives |
|  | Antidiarrheals, intestinal antiinflammatory / Antiinfective Agents | Intestinal antiinflammatory agents | Aminosalicyclic acid and similar agents |
|  | Bile and liver therapy | Bile Therapy | Bile acids and derivatives |
|  | Digestives, incl enzymes | Digestives, incl enzymes | Digestives, incl enzymes |
|  | drugs for acid related disorders | Drugs for peptic ulcer and gastro-oesophageal reflux disease (GORD) | Proton pump inhibitors |
|  | drugs for acid related disorders | Drugs for peptic ulcer and gastro-oesophageal reflux disease (GORD) | H2-receptor antagonists |
|  | Drugs for constipation | drugs for constipation | contact laxatives |
|  | drugs for functional gastrointestinal disorders | Propulsives | Propulsives |
|  | Drugs used for acid related disorders | Drugs for peptic ulcer and gastro-oesophageal reflux disease (GORD) | Proton pump inhibitors |
|  | Drugs used in diabetes | Insulins and Analogues | Insulins and Analogues for injection, fast-acting |
|  | Drugs used in diabetes | Blood glucose lowering drugs, excl insulins | Sodium-glucose co-transporter 2 (SGLT2) Inhibitors |
|  | Drugs used in diabetes | Blood glucose lowering drugs, excl insulins | Glucagon-like ppeptide-1 (GLP-1) analogues |
|  | Drugs used in diabetes | Insulins and Analogues | Insulins and Analogues for injection, long-acting |
|  | Drugs used in diabetes | Blood glucose lowering drugs, excl insulins | sulphonylureas |
|  | Drugs used in diabetes | Insulins and Analogues | Insulins and Analogues for injection, intermediate-acting |
|  | Drugs used in diabetes | Blood glucose lowering drugs, excl insulins | Dipeptidyl peptidase 4 (DPP-4) inhibitors |
|  | Drugs used in diabetes | Blood glucose lowering drugs, excl insulins | Biguanides |
|  | Drugs used in diabetes | Insulins and Analogues | Insulins and Analogues for injection, intermediate- or long-acting combined with fast-acting |
|  | Drugs used in diabetes | Blood glucose lowering drugs, excl insulins | Thiazolidinediones |
|  | Drugs used in diabetes | Blood glucose lowering drugs, excl insulins | other blood glucose lowering drugs, excl insulins |
|  | Mineral Supplements | Calcium | Calcium |
|  | Mineral Supplements | Other mineral supplements | Magnesium |
|  | Mineral Supplements | Potassium | Potassium |
|  | Mineral Supplements | Other mineral supplements | sodium |
|  | Stomatological Preparations | Stomatological Preparations | Corticosteroids for local oral treatment |
|  | Stomatological Preparations | Stomatological Preparations | Antiinfectives and antiseptics for local oral treatment |
|  | vitamins | vita | Vitamin D and analogues |
|  | vitamins | Ascorbic Acid (Vitamin C), Incl combinations | Ascorbic acid (vitamin C), plain |
|  | vitamins | Vitamin A and D, incl combinations of the two | Vitamin D and analogues |
|  | vitamins | mulivitamins, combinations | multivitamins with minerals |
| Antiinfectives for systemic use | Antibacterials for systemic use | Macrolides, lincosamides and Streoptogramins | Macrolides |
|  | Antibacterials for systemic use | Macrolides, lincosamides and Streoptogramins | Lincosamides |
|  | Antibacterials for systemic use | Combinations of sulfonamides and trimethoprim inclu derivatives | sulfamethoxazole and trimethoprim |
|  | Antibacterials for systemic use | other antibacterials | imidazole derivatives |
|  | Antivirals for Systemic use | Direct acting antivirals | nucleoside and nucleotide reverse transcriptase inhibitors |
| Antineoplastic and Immunomodulating agents | antineoplastic agents | Plant alkaloids and other natural products | Colchicine derivatives |
|  | antineoplastic agents | Antimetabolites | Folic acid analogues |
|  | Immunosuppressants | Immunosuppressants | Calcineurin inhibitors |
|  | Immunosuppressants | Immunosuppressants | Other Immunosuppressant |
|  | Immunosuppressants | Immunosuppressants | Selective immunosuppressants |
| antiparasitic products, insecticides and repellents | antiprotozoals | Antimalarials | aminoquinolines |
|  | antiprotozoals | agents against amoebiasis and other protozoal diseases | nitroimidazole derivatives |
| Blood and Blood Forming Organs | antianemic preparations | Iron preparations | Iron bivalent, oral preparations |
|  | antianemic preparations | Vitamin B12 and folic acid | Folic acid and derivatives |
|  | antianemic preparations | Vitamin B12 and folic acid | Vitamin B12 (cyanocobalamin and analogues) |
|  | Antithrombic Agents | Antithrombic Agents | Platelet aggregation inhibitors excl. heparin |
|  | Antithrombic Agents | Antithrombic Agents | Heparin group |
|  | Antithrombic Agents | Antithrombic Agents | Vitamin K antagonists |
|  | Blood Substitutes and Perfusion solutions | IV solution additives | electrolyte solutions |
| Cardiovascular system | Agents Acting on the Renin-Angiotensin System | ACE inhibitors, plain | ACE inhibitors, plain |
|  | Agents Acting on the Renin-Angiotensin System | Angiotensin II Receptor Blockers (ARBs), plain | angiotensin II receptor blockers (ARBs), plain |
|  | antihypertensives | Arteriolar smooth muscle, agents acting on | hydrazinophthalazine derivatives |
|  | Beta blocking agents | Beta Blocking Agents | Beta blocking agents, selective |
|  | Beta blocking agents | Beta Blocking Agents | Alpha and beta blocking agents |
|  | Beta blocking agents | Beta Blocking Agents | Beta blocking agents, non-selective |
|  | Calcium channel blockers | Selective Calcium Channel Blockers with Mainly Vascular Effects | Dihydropyridine derivatives |
|  | Cardiac Therapy | Vasodilators used in cardiac diseases | Organic nitrates |
|  | Diuretics | High-ceiling diuretics | Sulfonamides, plain |
|  | Diuretics | Aldosterone antagonists and other potassium sparing agents | aldosterone antagonists |
|  | Lipid Modifying Agents | Lipid modifying agents, plain | HMG CoA reductase Inhibitors |
|  | Lipid Modifying Agents | Lipid modifying agents, plain | Other lipid modifying agents |
|  | vasoprotectives | Capillary stabilizing agents | bioflavonoids |
|  | vasoprotectives | Agents for treatment of hemorrhoids and anal fissures for topical use | musle relaxants |
| Dermatologicals | anti-acne preparations | Anti-acne preparations for topical use | antiinfectives for treatment of acne |
|  | antibiotics and chemotherapeutics for dermatological use | chemotherapeutics for topical use | other chemotherapeutics |
|  | antipsoriatics | antipsoriatics for topical use | other antipsoriatics for topical use |
|  | corticosteroids, dermatological preparations | corticosteroids , plain | corticosteroids, weak |
|  | Other dermatological preparations | other dermatological preparations | agents for dermatitis, excluding corticosteroids |
|  | Other dermatological preparations | Other Dermatological Preparations | Other ophthalmologicals |
| Genito Urinary System and Sex Hormones | Gynecological Antiinfectives and Antiseptics | Gynecological Antiinfectives and Antiseptics, excl combinations with corticosteroids | organic acids |
|  | Gynecological Antiinfectives and Antiseptics | Antiinfectives and antiseptics excl combinations with corticosteroids | Antibiotics |
|  | Gynecological Antiinfectives and Antiseptics | Antiinfectives and antiseptics excl combinations with corticosteroids | imidazole derivatives |
|  | Urologicals | Drugs used in benign prostatic hypertropy | alpha-adrenoceptor antagonists |
|  | Urologicals | Drugs used in benign prostatic hypertropy | Testosterone-5-alpha reductase inhibitors |
|  | Urologicals | Urologicals | Drugs for urinary frequency and incontinence |
| Musculo-skeletal system | Antigout preparations | Antigout Preparations | Preparations inhibiting uric acid production |
|  | Antigout preparations | Antigout Preparations | preparations with no effect on uric acid metabolism |
|  | antiinflammatory and antirheumatic products | Antiinflammatory and antirheumatic products, non-steroids | other antiinflammatory and antirheumatic agents, non-steriods |
|  | antiinflammatory and antirheumatic products | Antiinflammatory and antirheumatic products, non-steroids | Propionic acid derivatives |
|  | Muscle Relaxants | Muscle relaxants, centrally acting agents | Other centrally acting agents |
|  | Muscle Relaxants | Muscle relaxants, centrally acting agents | Ethers, chemically close to antihistamines |
| Nervous System | Analgesics | Other analgesics and antipyretics | Salicyclic acid and derivatives |
|  | Analgesics | Other analgesics and antipyretics | Gabapentinoids |
|  | Analgesics | Opioids | Natural opium alkaloids |
|  | Analgesics | Other analgesics and antipyretics | Anilides |
|  | Analgesics | Opioids | other opiods |
|  | Anti-parkinson Drugs | dopaminergic agents | dopa and dopa derivatives |
|  | Anti-parkinson Drugs | Dopa and dopa derivatives | levodopa and decarboxylase inhibitor |
|  | Anti-parkinson Drugs | dopaminergic agents | monoamine oxidase B inhibitors |
|  | Anti-parkinson Drugs | anticholinergic agents | tertiary amines |
|  | Antiepileptics | Antiepileptics | Carboxamide derivatives |
|  | Antiepileptics | Antiepileptics | Benzodiazepine derivatives |
|  | Antiepileptics | Antiepileptics | other antiepileptics |
|  | Antiepileptics | Antiepileptics | Hydantoin derivatives |
|  | Antiepileptics | Antiepileptics | Fatty acid derivatives |
|  | Other nervous system drugs | Antivertigo Preparations | Antivertigo Preparations |
|  | Other nervous system drugs | Parasympathomimetics | Choline esters |
|  | Other nervous system drugs | Parasympathomimetics | anticholinesterases |
|  | Psychoanaleptics | Antidepressants | Non-selective monoamine reuptake inhibitors |
|  | Psychoanaleptics | Anti-dementia drugs | anticholinesterases |
|  | Psychoanaleptics | Antidepressants | Selective serotonin reuptake inhibitors |
|  | Psychoanaleptics | Antidepressants | other antidepressants |
|  | Psycholeptics | Anxiolytics | Diphenylmethane derivatives |
|  | Psycholeptics | Hypnotics and sedatives | melatonin receptor agonists |
|  | Psycholeptics | Antipsychotics | diazepines, oxazepines, thizepines and oxepines |
| Respiratory System | Antihistamines for systemic use | Antihistamines for systemic use | substituted alkylamines |
|  | Antihistamines for systemic use | Antihistamines for systemic use | other antihistamines for systemic use |
|  | Cough and Cold Preparations | Expectorants, excl combinations with cough suppressants | mucolytics |
|  | Drugs for obstructive Airway Diseases | Other systemic drugs for obstructive airway diseases | Leukotriene receptor antagonists |
|  | Nasal preparations | Decongestants and other Nasal Preparations for Topical use | Corticosteriods |
| Sensory Organs | Ophthalmologicals | Other ophthalmologicals | Other ophthalmologicals |
|  | Ophthalmologicals | Antiinflammatory agents | Corticosteriods |
| Systemic Hormonal Presparations, Excl sex hormones and insulins | Corticosteriods for systemic use | Corticosteroids for systemic use, plain | glucocorticoids |
|  | Thyroid Therapy | Antithyroid Preparations | Sulfur-containing imidazole derivatives |
|  | Thyroid Therapy | Thyroid preparations | Thyroid hormones |
| Various | All other therapeutic products | all other therapeutic products | Antidotes |
|  | All other therapeutic products | all other therapeutic products | Drugs for treatment of hyperkalemia and hyperphosphatemia |
