# Supplementary material for: Development and evaluation of a lightweight large language model chatbot for medication enquiry
Source: PLOS Digit Health. 2025 Sep 4;4(9):e0000961. doi: 10.1371/journal.pdig.0000961 (PMC12410746; doi:10.1371/journal.pdig.0000961)
Supplement: S3 Table — Validation and test questions were obtained from various open-sourced, online patient forums and portals. Questions were used in their original state without amendment or correction. (DOCX) [file pdig.0000961.s003.docx]

S3 Table. Validation and test questions data sources. Validation and test questions were obtained from various open-sourced, online patient forums and portals. Questions were used in their original state without amendment or correction.

| S/No | Source |
| --- | --- |
| 1 | <https://patient.info/forums/discuss/overwhelmed-694789> |
| 2 | <https://www.webmd.com/drugs/2/index> |
| 3 | <https://www.drugs.com/> |
| 4 | <https://www.askapatient.com/> |
