# Supplementary material for: Development and evaluation of a lightweight large language model chatbot for medication enquiry
Source: PLOS Digit Health. 2025 Sep 4;4(9):e0000961. doi: 10.1371/journal.pdig.0000961 (PMC12410746; doi:10.1371/journal.pdig.0000961)
Supplement: S4 Table — Validation and test questions can be characterised according to level of difficulty, question type and ATC categories of medications mentioned in the questions. (DOCX) [file pdig.0000961.s004.docx]

S4 Table: Example of validation and test questions. Validation and test questions can be characterised according to level of difficulty, question type and ATC categories of medications mentioned in the questions.
