# Supplementary material for: Development and evaluation of a lightweight large language model chatbot for medication enquiry
Source: PLOS Digit Health. 2025 Sep 4;4(9):e0000961. doi: 10.1371/journal.pdig.0000961 (PMC12410746; doi:10.1371/journal.pdig.0000961)
Supplement: S1 Fig — The test dataset contained a higher proportion of medium and high difficulty questions despite smaller sample size of the test dataset. (DOCX) [file pdig.0000961.s005.docx]

S1 Fig: Difficulty level of validation and test questions. The test dataset contained a higher proportion of medium and high difficulty questions despite smaller sample size of the test dataset.
